# Plasmablastic lymphoma with MYC::IGH fusion and BCL2 rearrangement

**DOI:** 10.1002/jha2.513

**Published:** 2022-06-15

**Authors:** Mohammad Barouqa, Patricia Greipp, Rebecca King, Ellen D. McPhail

**Affiliations:** ^1^ Mayo Clinic Department of Laboratory Medicine and Pathology Rochester Minnesota USA

1

A 50‐year‐old male with history of nodal stage III diffuse large B‐cell lymphoma (DLBCL), refractory to R‐CHOP, presented with vomiting, abdominal pain, and an umbilical mass. Abdominal computed tomography (CT) scan showed bulky retroperitoneal adenopathy and possible bowel obstruction. Histologic examination of the umbilical mass needle biopsy demonstrated diffuse atypical cells with medium to large nuclei, prominent central nucleoli, and eccentrically placed amphophilic cytoplasm (Figure 1, top left panel, objective 100×). The original lymphoma was of germinal center B‐cell phenotype, was MYC‐negative by immunohistochemistry (IHC), and had *BCL2* rearrangement (*BCL2*‐R) without *MYC*‐R by interphase fluorescence in situ hybridization (FISH). In contrast, the umbilical mass neoplastic cells expressed plasma cell markers CD138 (Figure 1, top right, objective 60×) and MUM1 (Figure 1. Bottom left, objective 60×) and MYC (90%), were lambda light chain restricted, had a very high Ki‐67 proliferation index (>90%), and were negative for all B‐cell markers, germinal center cell markers, and Epstein‐Barr virus. Interphase FISH studies identified *BCL2*‐R and *IGH::MYC* fusion (Figure 1, bottom right, Ch 8 centromere: aqua probe, MYC [8q24]: red probe, IGH [14q32]: green probe; yellow fusion indicates *IGH::MYC*) without *BCL6*‐R. These findings are characteristic of plasmablastic lymphoma with *MYC::IGH* fusion and *BCL2*‐R. This likely represents transformation of the patient's prior DLBCL, with clonal evolution characterized by acquisition of *MYC*‐R. Transformation of follicular lymphoma to plasmablastic lymphoma with *MYC*‐R and *BCL2*‐R has been previously described, but to our knowledge this is the first reported case of transformation of DLBCL to plasmablastic lymphoma with *MYC::IGH* fusion and *BCL2*‐R. The “double‐hit” cytogenetic features may portend an aggressive clinical course, similar to what has been observed in “high‐grade B‐cell lymphomas with *MYC*‐R and *BCL2*‐R and/or *BCL6*‐R” (so‐called double‐hit lymphoma).

**FIGURE 1:**
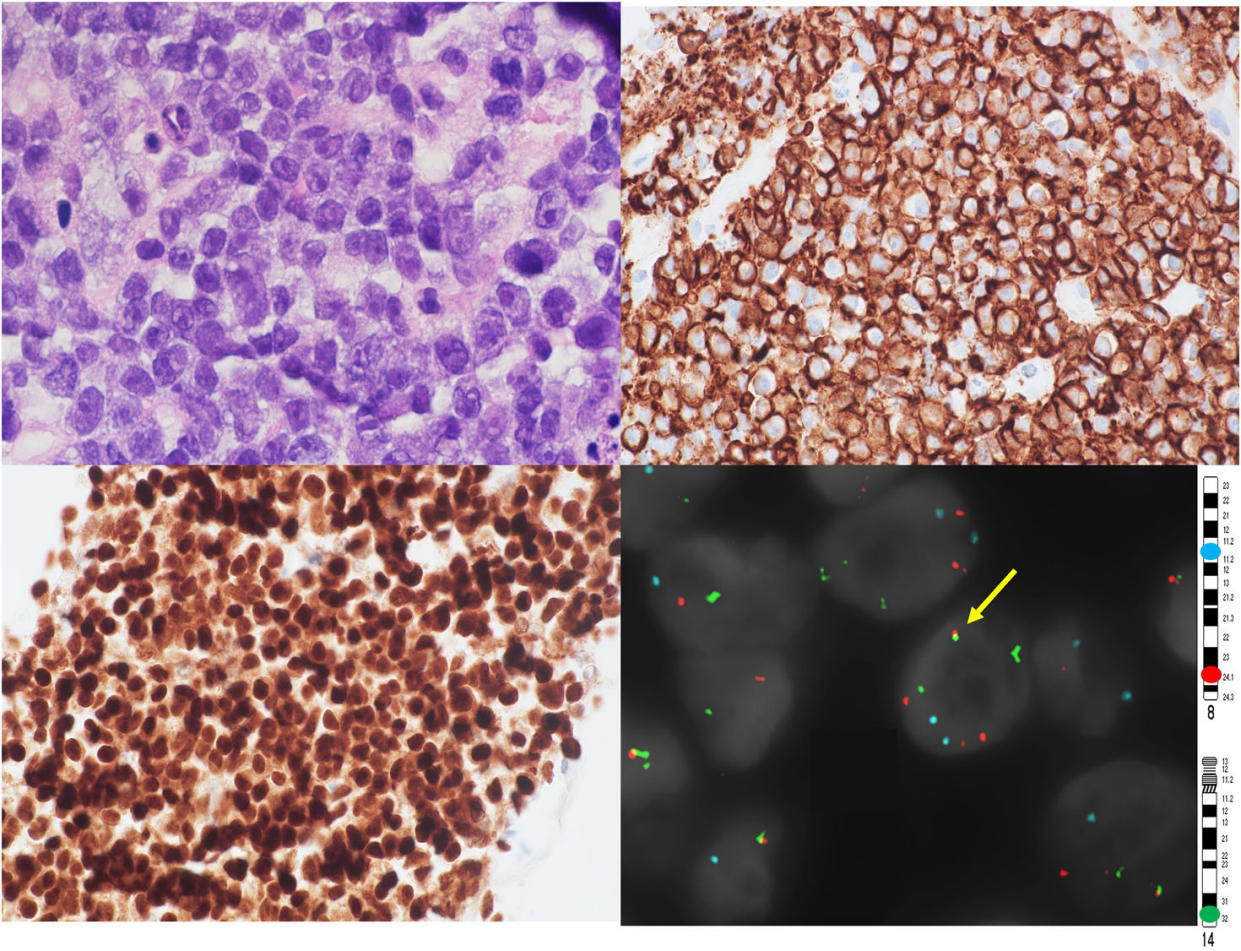
Top left: H&E stain. Top right: CD138 stain. Bottom left: MUM‐1 stain. Bottom right: Fluorescence in situ hybridization (FISH) study

## CONFLICT OF INTEREST

The authors declare they have no conflict of interest.

## FUNDING INFORMATION

The authors received no specific funding for this work.

## AUTHOR CONTRIBUTIONS

Mohammad Barouqa wrote the manuscript. Patricia Greipp reviewed the article and cytogenetic probes. Rebecca King reviewed the article and stains. Ellen D. McPhail supervised the manuscript and finalized it.

